# Supplementary material for: Dynamic capillary assembly of colloids at interfaces with 10,000g accelerations
Source: Nat Commun. 2018 Sep 6;9:3620. doi: 10.1038/s41467-018-06049-9 (PMC6127265; doi:10.1038/s41467-018-06049-9)
Supplement: Supplementary file 1 — Supplementary Information [file 41467_2018_6049_MOESM1_ESM.pdf]

SUPPLEMENTARY INFORMATION
Dynamic capillary assembly of colloids at interfaces with 10,000g accelerations

Axel Huerre, Marco De Corato, and Valeria Garbin

Department of Chemical Engineering, Imperial College London, UK.

Contents

Supplementary Note 1: Data analysis 2

1.1 Tracking of bubble radius . . . 2

1.2 Detection of particle centres . . . 2

1.3 Calculation of surface coverage . . . 3

1.4 Pair correlation function and definition of neighbours . . . 3

1.5 Bond order parameters Ψ_m . . . 4

1.6 Definition of the network of strings . . . 4

1.7 Definition of the Weber number . . . 5

Supplementary Note 2: Harmonic oscillator model 6

Supplementary Note 3: Interaction model 7

3.1 Overview of the model . . . 7

3.2 Capillary interactions . . . 8

3.3 Lateral hydrodynamic interactions . . . 11

3.4 Excluded volume interactions . . . 12

3.5 Surface constraining force . . . 12

3.6 Governing equations . . . 12

3.7 Effect of individual interactions on the microstructure . . . 15

3.8 Relaxation of the microstructure . . . 16

Supplementary Figure 1 18

Supplementary Figure 2 19

Supplementary Figure 3 20

Supplementary Figure 4 21

Supplementary Figure 5 22

Supplementary Figure 6 23

Supplementary Figure 7 24

Supplementary Figure 8 25

Supplementary Figure 11 26

Supplementary Figure 9 27

Supplementary Figure 10 28

Supplementary References 29

Supplementary Note 1: Data analysis

1.1 Tracking of bubble radius

The initial value of the bubble radius is measured manually with ImageJ by drawing a circle fitting with the contour, measuring the corresponding area and converting it in an initial radius R_0. Then, the picture is converted to black and white with MATLAB function im2bw, adjusting the threshold to recover R_0. The threshold is then kept constant for the whole video analysis. For each frame, after conversion, we detect the contour points with the function bwboundaries and finally fit the minimal bounding circle to find the bubble radius $R(t)$. From this, we can define the radial excursion. We took a sliding window of 15 frames (2 cycles at 40 kHz excitation and 300,000 fps) and find the minimum R_- and maximum R_+ radii in this time window. We then define $\delta R(t) = (R_+ - R_-)/2$. The curve $\delta R(t)$ plateaus after a transient time. Experimentally, we found that this transient time is on the order of 30 cycles. We finally are able to compute the radial excursion ΔR as the mean value of $\delta R(t)$ for times greater than 35 cycles and the error made on it $d\Delta R$ as the standard deviation.

1.2 Detection of particle centres

We used semi-automated particle detection. The first step consists of a manual recording of particles position to give a rough estimate of the centres. The second step allows us to obtain sub-pixel accuracy. Rather than using the classical Gaussian fitting method, we implemented a method developed by Parthasarathy [1] based on an analytic calculation of the best-fit radial symmetry centre. The manually-obtained data are fed in the sub-pixel detection algorithm. We then calculate around each of the centres (a window is taken around the centre with height and width being $2a + 2$ pixels, with a the particle radius) the point of minimal distance to the intensity gradient lines. This method is fast and gives the coordinates of a centre with a precision smaller than 0.1 pixel (with a SNR=6.4 in our experiment, the theoretical precision is 2×10^{-2} pixels). Figure 1a shows the resulting coordinates (yellow crosses) after treatment by the algorithm of the manually detected centres (red dots). Another source of error in the particle centre detection is the blur linked to the interface movement. The magnitude of the velocity of a particle on the interface can be evaluated as $V = \Delta R\omega \approx 0.3$ m s^{-1}. With the high-speed camera recording at $f_{\mathrm{rec}} = 300,000$ fps, the particle can move a distance $\Delta x \approx V/f_{\mathrm{rec}} = 1\,\mu$m $= 1$ pixel. The main error in particle position is therefore due to image blur. However, looking mainly at particles at the bubble pole (with a radial velocity pointing toward the camera), the projected V is greatly reduced, and the error of measurement is under a pixel.

1.3 Calculation of surface coverage

Once the centres of the particles are detected, we can compute the surface coverage Φ of particles at the interface, defined as

$$\Phi = \frac{N\pi a^2}{A}\,, \tag{1}$$

where N is the number of detected particles of radius a, and A is the area of detection. Because we are not able to measure a large number of particles, we have to be careful with the definition of the area of measurement. We compute two extreme values for this area, being the area of the convex hull A_{h} (minimum area enclosing all the particles) and the area of the minimum bounding circle A_{c} (area of the circle of minimal radius enclosing all the particles). As seen in Supplementary Figure 1b, the area of detection can be important relatively to the bubble area and thus, the directly measured area can be different form the real one occupied by the particles. To take this into account, we correct the measured area $A_{\mathrm{c,h}} = \pi l_{\mathrm{c,h}}^2$ by the corresponding spherical cap area:

$$A_{\mathrm{c,h,cap}} = 2\pi R_0 (R_0 - \sqrt{R_0^2 - l_{\mathrm{c,h}}^2})\,, \tag{2}$$

with R_0 the bubble's radius. These two values allow us to correct for the spherical cap and we can compute the two extreme particle densities:

$$\Phi_{\mathrm{c}} = \frac{N\pi a^2}{A_{\mathrm{c,cap}}} \quad \text{and} \quad \Phi_{\mathrm{h}} = \frac{N\pi a^2}{A_{\mathrm{h,cap}}}\,. \tag{3}$$

We finally define the particle density Φ and the experimental error $\Delta\Phi$

$$\Phi = \frac{\Phi_{\mathrm{c}} + \Phi_{\mathrm{h}}}{2} \quad \text{and} \quad \Delta\Phi = \frac{|\Phi_{\mathrm{c}} - \Phi_{\mathrm{h}}|}{2}\,. \tag{4}$$

1.4 Pair correlation function and definition of neighbours

The radial distribution or pair correlation function is calculated as

$$g(r) = \frac{1}{\Phi \cdot 2\pi r \Delta r \cdot (N-1)} \sum_{j \neq i} \delta(r - |\mathbf{r}_{ij}|)\,, \tag{5}$$

where N is the total number of particles detected, Δr is the increment of r and $|\mathbf{r}_{ij}|$ is the centre-to-centre distance between particles i and j. From this calculation, we set a distance criterion for the definition of a particle's neighbours. The latter are defined as being at a distance smaller than the first minimum of the correlation function in the initial state. For our experiments, this results in the following criterion:

$$\text{particle } k \text{ is a neighbour of particle } j \text{ if } |\mathbf{r}_{ij}| \leq 2.5a. \tag{6}$$

This definition accounts for errors in particle center detection, and includes all particles which fall in the first peak of $g(r)$. The probability of having a number of neighbour equal to n is then defined as:

$$p(n) = \frac{N_n}{N}\,, \tag{7}$$

with N_n the number of particles having n neighbours. We then compute the mean number of neighbours per particle, $\bar{n} = \sum_{k=0}^{6} kp(k)$.

1.5 Bond order parameters Ψ_m

A simple mean of characterising the local structural order in a 2D ordered network is the use of bond order parameters defined as:

$$\psi_m^i = \frac{1}{n_i}\sum_{j=1}^{n_i} \exp^{\sqrt{-1}\,m\,\alpha_{jk}}\,, \tag{8}$$

with n_i the number of neighbours of particle i and α_{ij} the angle between a reference vector (we used the centre-to-centre vector with the closest neighbour) and the centre-to-centre vector $\mathbf{r}_{ij}$. Usually, the hexatic ($m = 6$) parameter is computed to compare the network of particles with an hexagonal packing. As we are interested in characterising a network of strings, we define the 2-fold and 3-fold bond order parameters ($m = 2$; $m = 3$ respectively). We can then compute for the entire network the mean bond order parameters:

$$|\Psi_m| = \frac{1}{N}\sum_{i=1}^{N} \psi_m^i\,, \tag{9}$$

with N the number of particles in the network. $|\Psi_2| = 1$ means that each particle has two neighbours, and that the particle and neighbours are aligned (see Supplementary Figure 3a). $|\Psi_3| = 1$ means that the particles have three neighbours that respect the sp2 geometry (equilateral triangle, see Supplementary Figure 3b). More complex networks including branched strings show intermediate values of $|\Psi_2|$ and $|\Psi_3|$ as shown in Supplementary Figure 3c.

1.6 Definition of the network of strings

In the majority of the experiments and simulations, strings could be easily identified by the naked eye. However, to dissipate any doubt, we defined the following set of quantitative criteria to consistently identify strings in experiments and simulations:

1. $g\,(r/a = 4) > 1$ and $g(r/a = 4) > g(r/a = 3.5)$;

2. $p(n=2,3) > p(n=4,5,6)$.

1.7 Definition of the Weber number

We define the Weber number, We, based on the velocity of the interface $\dot{R}$, as:

$$We = \frac{\Delta\rho a^2 \dot{R}^2}{\gamma \Delta R}. \tag{10}$$

We can recast We to highlight the similarity with the Bond number, $Bo = \frac{\Delta\rho g a^2}{\gamma}$, that is used to evaluate the presence of a monopolar deformation due to gravitational effects, using $\dot{R} = \omega\,\Delta R$ and $\ddot{R} = \omega^2\,\Delta R$:

$$We = \frac{\Delta\rho a^2 \omega^2 \Delta R^2}{\gamma \Delta R} = \frac{\Delta\rho a^2 \ddot{R}}{\gamma}. \tag{11}$$

The Weber number based on the kinetic energy returns the same expression because in our system $a/\Delta R \sim 1$.

The following approximations are made for the constants in the calculation of all dimensionless numbers: we consider $\Delta\rho \approx \rho$, where ρ is the density of the liquid, because the density of the polystyrene particles is similar to that of water, and both are much larger than the density of air: $\rho_{\mathrm{p}} \sim \rho \gg \rho_{\mathrm{air}}$. Note that the relevant density difference is that between the particle and air, and not that between the particle and the liquid, because part of the colloids is immersed in air and this contribution is dominant. The surface tension is set to $\gamma = 35$ mN m^{-1} to effectively account for the presence of the monolayer of particles.

The error made in estimating the Weber number, neglecting the relative errors on frequency, density and surface tension, is given by

$$dWe = We\left(\frac{d\Delta R}{\Delta R} + 2\frac{da}{a}\right). \tag{12}$$

The first term is usually 2-3%, and the second term depends strongly on the size of the particles: 2.6%, 4%, 5.3%, and 14.5% for $a = 0.9$, 1.5, 2.5 and 5 μm, respectively.

Supplementary Note 2: Harmonic oscillator model

We write the normal force balance on a particle, in a reference frame moving with the interface as $R(t) = R_0 + \Delta R \sin(\omega t)$. We consider the contact line to be pinned, so that a displacement of the particle normal to the interface causes the formation of a capillary bridge (see Supplementary Figure 6a). The resulting restoring force is modeled by Hooke's law, where the stiffness of the spring is given by the surface tension, γ [2]. We assume the contact contact angle to be $\frac{\pi}{2}$. The mass of the harmonic oscillator is taken for simplicity to be the mass of the particle. We also include in the force balance the viscous drag on the particle, assuming that it is given by half of the Stokes drag for a particle that is half immersed in water. The force driving the harmonic oscillator is given by the fictitious force acting on the particle due the sinusoidal acceleration of the reference frame that is fixed to the bubble surface (see Supplementary Figure 6b). We neglect the effects of air density and viscosity, and neglect the density difference between polystyrene and water, $\rho_\mathrm{p} \sim \rho \gg \rho_\mathrm{air}$, so that $\Delta\rho \approx \rho$, where ρ is the density of water.

The dimensionless force balance on the particle is given by:

$$\frac{4\pi}{3} We \frac{a}{\Delta R} \ddot{x} + 3\pi\, Ca\, \dot{x} + x = \frac{2\pi}{3}\, We \, \sin(t)\,, \tag{13}$$

where we have chosen the particle radius a as characteristic length, and ω^{-1} as characteristic time. In Equation (13) We is the Weber number, defined previously, and $Ca = \dfrac{\eta\, a\omega}{\gamma}$ the capillary number, with η the viscosity of water.

The solution of Equation (13) can be written as $x = \dfrac{Q_0\,(We, Ca)}{a} \sin(t + \phi)$, where $Q_0\,(We, Ca)$ represents the amplitude of the time-dependent monopolar deformation of the interface.

In most of the experiments, $\Delta R \approx a$, $Ca \approx 10^{-3} - 10^{-2}$, and $We \approx 10^{-3} - 10^{-1}$. The oscillator is underdamped, and the forcing frequency is much smaller than the resonance frequency. As a consequence, the damping term has little effect on the dynamics of the particle, which is only slightly out of phase with the driving force ($\phi \approx 0$). A good estimate of the amplitude of the vertical deformation in typical experimental conditions is $Q_0\,(We)\,/a \approx \frac{2\pi}{3}\, We$.

Supplementary Note 3: Interaction model

3.1 Overview of the model

In this section we present a simplified model that describes the dynamics of the particles on the surface of the bubble. Direct numerical simulations of the dynamic deformation field of the spherical bubble induced by the ultrasound forcing and by the forces acting on all the particles is cumbersome, if at all possible. Hence, we developed a simplified model for capillary and hydrodynamic forces, in which particles interact in a pair-wise fashion. This model captures the physical mechanisms responsible for the formation of strings in the experiments.

We assume that the deformation of the bubble surface induced by each particle is small and can be decomposed into two principal multipolar contributions [3]: a monopolar and a quadrupolar term. Higher-order multipoles have been neglected for simplicity, because the experimental findings are captured to a sufficient level of detail by this minimal model. By neglecting multibody interactions, the capillary forces and torques acting between the particles are given by analytical formulas [4, 5]. In addition to capillary interactions, we include in the model the lateral hydrodynamic force due to the flow fields generated by the vertical periodic motion of the other particles.

We consider a set of N spherical particles of radius a confined to a spherical surface of radius $R(t)$. We assume that the spherical surface evolves with a periodic function of time $R(t) = R_0 + \Delta\, R\, \sin(\omega\, t)$, and we define the position vector of particle i as $\mathbf{r}_i(t)$ (see Supplementary Figure 11a). The surface area fraction occupied by the adsorbed particles is given by $\Phi = \frac{N\, a^2}{4R_0^2}$.

As discussed in Sec. 2, a particle i moves relative to the interface in the radial direction due to its inertia. The periodic sink/rise of a particle results in a time-dependent capillary monopole [5], the amplitude of which is assumed to be:

$$Q_0^i(t) = Q_0^i\, \sin(\omega\, t)\,. \tag{14}$$

The amplitude Q_0^i is chosen consistently with the prediction of the harmonic oscillator model (see Sec. 2) and is assumed to be the same for all the particles. This is equivalent to assuming that the particles are monodisperse and all have the same contact angle. An example of monopolar deformation of an interface is depicted in Supplementary Figure 11b.

We assume that the contact line of each particle is undulated due to nanoscale roughness of the surface of the particle [6, 7] and is described by a permanent quadrupole of amplitude Q_2^i, as shown in Supplementary Figure 11c. For simplicity, we consider Q_2^i to be the same for all the particles. This quantity may also be polydisperse in experiments, due to the variability in contact line undulations

between different particles. A capillary quadrupole is completely determined by its amplitude Q_2^i, and by an orientation vector $\mathbf{p}_i(t)$, which is tangent to the undeformed interface, as shown schematically in Supplementary Figure 11a. In other words, $\mathbf{p}_i(t)$ is a vector tangent to the bubble surface at each time t and describes the direction of the elevated part of contact line (see Supplementary Figure 11c).

The overlap of interfacial deformations drives pair-wise lateral capillary forces and torques between the particles. Three capillary interactions are considered:

- A dynamic monopole $Q_0^i(t)$ interacting with a dynamic monopole $Q_0^j(t)$.
- A permanent quadrupole Q_2^i interacting with a static quadrupole Q_2^j.
- A static quadrupole Q_2^iinteracting with a dynamic monopole $Q_0^j(t)$, and vice versa.

The equations for the lateral capillary forces and torques are given in Section 3.2. The model for the hydrodynamic interactions between particles due to their vertical periodic motion is presented in Section 3.3.

3.2 Capillary interactions

Monopole-monopole interaction

The lateral capillary force on particle i due to the interaction of its monopolar deformation $Q_0^i(t)$ with the monopolar deformation $Q_0^j(t)$ induced by particle j (Supplementary Figure 5a) is given by [5]:

$$\mathbf{F}_{00}^{ij} = \frac{\gamma \, Q_0^i(t) \, Q_0^j(t)}{2 \, \pi \, d} \, \hat{\mathbf{d}}_{ij} \, . \tag{15}$$

In the equation above, γ is the surface tension, $Q_0^i(t)$ and $Q_0^j(t)$ are given by Eq. (14), and d is the distance on the bubble surface between particle i and particle j (see Supplementary Figure 5a). d is defined as:

$$d = \arccos\left(\frac{\mathbf{r}_i \cdot \mathbf{r}_j}{|\mathbf{r}_i||\mathbf{r}_j|}\right) R(t) \, . \tag{16}$$

In Equation (15), the vector $\hat{\mathbf{d}}_{ij}$ is a unit vector with its origin at the center of particle i, directed from particle i to particle j, and tangent to the bubble surface as shown schematically in Supplementary Figure 11a. It is defined as:

$$\hat{\mathbf{d}}_{ij} = \frac{(\mathbf{r_j} - \mathbf{r_i}) \cdot \left(\mathbf{I} - \frac{\mathbf{r}_i \mathbf{r}_i}{\mathbf{r}_i \cdot \mathbf{r}_i}\right)}{\left|(\mathbf{r_j} - \mathbf{r_i}) \cdot \left(\mathbf{I} - \frac{\mathbf{r}_i \mathbf{r}_i}{\mathbf{r}_i \cdot \mathbf{r}_i}\right)\right|} \, . \tag{17}$$

Equation (15) describes the interaction force between two monopoles on a planar interface, which is always attractive, independent of their positions on the interface. Dynamical effects, which are present in the experiments but are not included in the model, could impact the radial dependence of the deformation, but do not affect its axisymmetry.

Quadrupole-quadrupole interaction

The capillary force and torque on particle i due to the interaction of its quadrupolar deformation Q_2^i with the quadrupolar deformation Q_2^j induced by particle j depend on the interparticle distance and on the relative orientations of the quadrupoles. We assume that the lateral capillary force acting on particle i is given by [8]:

$$\mathbf{F}_{22}^{ij} = \frac{48\,\pi\,\gamma\,a^4\,Q_2^i\,Q_2^j}{d^5}\left[\hat{\mathbf{d}}_{ij}\cos\left(2\varphi_i + 2\varphi_j\right) - \frac{\mathbf{r}_i}{|\mathbf{r}_i|} \times \hat{\mathbf{d}}_{ij}\sin\left(2\varphi_i + 2\varphi_j\right)\right], \tag{18}$$

where Q_2^i and Q_2^j are the amplitudes of the quadrupolar modes of the contact line deformations on particle i and particle j, respectively. The angles θ_i and θ_j are schematically depicted in Supplementary Figure 5b. They are defined in terms of the particle positions $\mathbf{r}_{i,j}$ and orientation vectors $\mathbf{p}_{i,j}$ as:

$$\varphi_i = \text{atan2}\left(\frac{\mathbf{r}_i}{|\mathbf{r}_i|}\cdot\mathbf{p}_i \times \hat{\mathbf{d}}_{ij}\ ,\ \left(\mathbf{p}_i \cdot \hat{\mathbf{d}}_{ij}\right)\right), \tag{19}$$

$$\varphi_j = \text{atan2}\left(\frac{\mathbf{r}_j}{|\mathbf{r}_j|}\cdot\mathbf{p}_j \times \hat{\mathbf{d}}_{ji}\ ,\ \left(\mathbf{p}_j \cdot \hat{\mathbf{d}}_{ji}\right)\right), \tag{20}$$

with $\hat{\mathbf{d}}_{ji}$ shown schematically in Supplementary Figure 11a, and defined as:

$$\hat{\mathbf{d}}_{ji} = \frac{(\mathbf{r_j} - \mathbf{r_i})\cdot\left(\mathbf{I} - \frac{\mathbf{r}_j\mathbf{r}_j}{\mathbf{r}_j\cdot\mathbf{r}_j}\right)}{\left|(\mathbf{r_j} - \mathbf{r_i})\cdot\left(\mathbf{I} - \frac{\mathbf{r}_j\mathbf{r}_j}{\mathbf{r}_j\cdot\mathbf{r}_j}\right)\right|}. \tag{21}$$

The first term in Equation (18) is directed along the center-to-center vector. The second term is a force in the direction perpendicular to the center-to-center vector. Both terms are tangential to the spherical surface.

The capillary torque on the particle i is given by:

$$\mathbf{T}_{22}^{ij} = -\frac{24\,\pi\,\gamma\,a^4\,Q_2^i\,Q_2^j}{d^4}\frac{\mathbf{r}_i}{|\mathbf{r}_i|}\sin\left(2\varphi_i + 2\varphi_j\right). \tag{22}$$

The capillary torque is normal to the bubble surface (directed along $\mathbf{r}_i$) so that two particles will rotate until their orientation vectors respect $\varphi_i = -\varphi_j$ for which the capillary torque is zero.

Equations (18) and (22) state that the interactions between two quadrupoles is the same as that experienced by two particles on a flat interface, with the angles φ_i and φ_j computed on the planes tangent to the spherical surface, and the distance d evaluated along the bubble surface.

Monopole-quadrupole interaction

The monopolar deformation induced by particle i overlaps with the quadrupolar deformation of particle j, giving rise to a lateral capillary force given by [8]:

$$\mathbf{F}_{02}^{ij} = \frac{2\,\gamma\,a^2\,Q_0^i(t)\,Q_2^j}{d^3}\left[\hat{\mathbf{d}}_{ij}\cos\left(2\varphi_j\right) - \frac{\mathbf{r}_i}{|\mathbf{r}_i|}\times\hat{\mathbf{d}}_{ij}\sin\left(2\varphi_j\right)\right]\,. \tag{23}$$

Likewise, the quadrupolar deformation induced by particle i overlaps with the monopolar deformation of particle j, giving rise to a lateral capillary force and a torque given by [8]:

$$\mathbf{F}_{20}^{ij} = \frac{2\,\gamma\,a^2\,Q_2^i\,Q_0^j(t)}{d^3}\left[\hat{\mathbf{d}}_{ij}\cos\left(2\varphi_i\right) - \frac{\mathbf{r}_i}{|\mathbf{r}_i|}\times\hat{\mathbf{d}}_{ij}\sin\left(2\varphi_i\right)\right]\,, \tag{24}$$

$$\mathbf{T}_{20}^{ij} = -\frac{2\,\gamma\,a^2\,Q_2^i\,Q_0^j(t)}{d^2}\frac{\mathbf{r}_i}{|\mathbf{r}_i|}\sin\left(2\varphi_i\right). \tag{25}$$

Equation (25) implies that particle i will experience a torque until its orientation vector becomes parallel or perpendicular to the center-to-center vector ($\varphi_i = 0 \pm \pi$ or $\varphi_i = \frac{\pi}{2} \pm \pi$). Furthermore, interactions arising from the overlap of monopolar and quadrupolar deformations of the interface (Equations (23)-(25)) only allow particles to approach each other if the orientation vector is aligned or perpendicular to the center to center vector. As a consequence, this capillary interaction promotes the formation of strings of particles with their orientation vectors aligned.

Conversely, the interactions given by the overlap of two quadrupolar deformations (Equations (18)-(22)) favour the approach of particles having parallel orientation vectors ($\varphi_i = -\varphi_j$), thus promoting packing of particles in hexagonal or square arrays of aligned particles, as observed in literature both experimentally and by means of numerical simulations [9, 10]. The same observation applies for the overlapping of monopolar deformations [5].

Timescale for string formation

In the experiments and simulations, strings appear after a few hundreds of cycles of oscillations, as can be seen from the evolution of the order parameters $|\Psi_2|$ and $|\Psi_3|$ (Fig. 2f in the main text), which increase significantly during the initial 250 cycles. The microstructure is then sustained by the periodic forcing, as can be seen in Figure 1b of the main text, and as confirmed by the order parameters, which remain approximately constant for $N_\mathrm{c} > 250$. The slow dynamics of emergence of strings, over a timescale $\tau \gg 2\pi/\omega$, can be understood by balancing the mean force over one period of oscillation, $\langle F_{02}\rangle_T \propto -\frac{3}{2}\gamma a^2 Q_0 Q_2 \frac{1}{d_0^3}\frac{\Delta d}{d_0}$, with the translational drag on a particle at the interface, $\zeta_\mathrm{t} \approx 3\pi\eta a V$, with V the characteristic velocity. Note that the drag force is approximated with half of Stokes' drag because

the particle is at a liquid-gas interface. The timescale for a particle to translate over a distance equal to its radius a is given by $\tau_{t,02} \approx \frac{16\pi a^3 \eta}{\gamma Q_0 Q_2} \approx 5$ ms. This is to be compared to the period of oscillations, which for forcing at a frequency $f = \omega/2\pi = 40$ kHz is $\tau = 25\,\mu$s, consistent with a slow evolution over 200 cycles.

3.3 Lateral hydrodynamic interactions

In Sec 2, we showed that under typical experimental conditions each particle experiences a vertical periodic displacement relative to the interface $x \approx Q_0/a \sin(t)$. Supplementary Figure 6c shows that expected values for the vertical displacements are $\frac{Q_0}{a} \approx 0.01 - 0.5$. Thus, each particle is undergoing a small-amplitude and time-reversible vertical motion, which gives rise to velocity and pressure disturbances in the liquid. Nearby particles interact with these flow and pressure disturbances, experiencing a lateral hydrodynamic force.

The Reynolds number related to the motion of the particles is $Re = \frac{\rho\, a^2 \omega}{\eta} \approx 1$. Despite the micrometric size of the particles, inertial effects must be considered to describe the flow around each particle. For non-zero Reynolds number, a particle undergoing small-amplitude and time reversible oscillations in bulk generates a *steady* flow field, which is known as streaming flow [11, 12, 13]. Experimental and theoretical investigations of the dynamics of millimetric particles oscillating near a wall have shown that the near-field hydrodynamic forces due to the streaming flows are repulsive when the separation distance is similar to the viscous boundary layer's thickness [14, 15, 16]. In typical experiments, the boundary layer's thickness $\sqrt{\frac{2\,\eta}{\rho\,\omega}} \approx 2.5\,\mu$m is comparable to the size of the particle and to typical interparticle distances. Thus, we expect lateral repulsive hydrodynamic forces to act between the particles (see Supplementary Figure 7). However, the conditions met in the experiments are not described by those theoretical works, as all of the works focused on particle oscillating in the bulk of a fluid. Consequently, we can only discuss the expected magnitude and the scaling of the lateral forces due to streaming flows and propose an empirical model for this repulsive interaction supported by previous experimental works [17, 18].

In the limit of small vertical displacements, $Q_0 \ll a$, the magnitude of the streaming flow around the particle scales as $v \propto \left(\frac{Q_0}{a}\right)^2 a\,\omega$ [11]. Thus, the magnitude of the lateral hydrodynamic force can be estimated as $F_{\text{hyd}} \propto \beta \left(\frac{Q_0}{a}\right)^2 \eta\, a^2\, \omega$, where the numerical prefactor β is unknown. As $v \ll a\omega$, the lateral motion of the particle is expected to be in the Stokes regime and β is thus expected to be comparable to 6π, as it is for particles moving in the bulk.

Moreover, assuming F_{hyd} to be proportional to the leading order term of the streaming velocity [11], we use a quadratic power law decay with the separation distance d. The repulsive hydrodynamic interaction between particles i and j is finally given by:

$$\mathbf{F}^{ij}_{\mathrm{hyd}} = -\beta \left(\frac{Q_0^j}{a}\right)^2 \eta\, a^2\, \omega\, \left(\frac{a}{d}\right)^2 \hat{\mathbf{d}}_{ij}\,. \tag{26}$$

3.4 Excluded volume interactions

To prevent particle overlapping in the simulations, we implement an excluded volume interaction between the colloids. It is set-up by means of a stiff spring that acts only when two particles intersect:

$$\mathbf{F}^{ij}_{\mathrm{EV}} = -2K_{\mathrm{EV}}\, \delta\, \hat{\mathbf{d}}_{ij} \qquad \text{if} \quad \delta < 0\,, \tag{27}$$

and

$$\mathbf{F}^{ij}_{\mathrm{EV}} = 0 \qquad \text{if} \quad \delta > 0\,, \tag{28}$$

where δ is the overlap between two particles, defined as $\delta = d - 2a$. The spring constant K_{EV} is chosen such that δ never exceeds $0.01a$, avoiding significant intersection between particles without stiffening the equations.

3.5 Surface constraining force

The particles are confined to the interface by a spring. The resulting constraining force that acts in the radial direction on each particle is:

$$\mathbf{F}^{i}_{\mathrm{S}} = -K_{\mathrm{S}}\,(|\mathbf{r}_i| - R(t))\, \frac{\mathbf{r}_i}{|\mathbf{r}_i|}\,, \tag{29}$$

with a spring constant K_{S} chosen such that the particles never move more than $0.05\,a$ away from the spherical surface.

3.6 Governing equations

With the capillary forces and torques given by Equations (15)-(25), the lateral hydrodynamic forces given by Equation (26), and the spring forces given by Equations (27)-(29), the evolution of the position and orientation of particle i is given by Newton's law:

$$\frac{4\pi}{3}\rho_{\mathrm{P}} a^3 \frac{d\mathbf{v}_i}{dt} = -3\pi\eta a \mathbf{v}_i + \mathbf{F}^{i}_{\mathrm{S}} + \sum_{j\neq i} \left(\mathbf{F}^{ij}_{00} + \mathbf{F}^{ij}_{22} + \mathbf{F}^{ij}_{02} + \mathbf{F}^{ij}_{20} + \mathbf{F}^{ij}_{\mathrm{EV}} + \mathbf{F}^{ij}_{\mathrm{hyd}}\right)\,, \tag{30}$$

$$\frac{d\mathbf{r}_i}{dt} = \mathbf{v}_i\,, \tag{31}$$

$$\frac{8\pi}{15}\rho_p a^5 \frac{d\boldsymbol{\omega}_i}{dt} = -4\pi\eta a^3 \boldsymbol{\omega}_i + \sum_{j\neq i}\left(\mathbf{T}_{22}^{ij} + \mathbf{T}_{20}^{ij}\right) , \tag{32}$$

$$\frac{d\mathbf{p}_i}{dt} = \boldsymbol{\omega}_i \times \mathbf{p}_i \, , \tag{33}$$

with η and ρ_p the viscosity of water and the density of the polystyrene particles, respectively; while $\mathbf{v}_i$ and $\boldsymbol{\omega}_i$ are the translational and rotational velocities of particle i, respectively. Since the lateral motion of the particles on the bubble surface is much slower than the vertical oscillations, we assume the translational and rotational friction coefficients of the particles to be equal to half of the value predicted by the Stokes law [19].

Making the equations of motion dimensionless by choosing the particle radius a as characteristic length, and the inverse of the bubble oscillation frequency ω^{-1} as characteristic time, we obtain the following dimensionless numbers:

St	Ca	Q_0^*	Q_2^*	ΔR^*	R_0^*	K_{EV}^*	K_{S}^*
$\frac{\rho_{\mathrm{P}} a^2 \omega}{\eta}$	$\frac{\eta\omega a}{\gamma}$	$\frac{Q_0}{a}$	$\frac{Q_2}{a}$	$\frac{\Delta R}{a}$	$\frac{R_0}{a}$	$\frac{K_{\mathrm{EV}}}{\eta\omega a}$	$\frac{K_{\mathrm{S}}}{\eta\omega a}$

Supplementary Table 1: Dimensionless parameters and their expressions.

The Stokes number St is the ratio between the inertia of the particle and the viscous force. Due to the fast deformation of the interface in the experiments, $St \approx 1$. The particle inertia and viscous forces are comparable in magnitude, thus in all the simulations we used $St = 1$.
The capillary number Ca represents the ratio between the viscous forces and the lateral forces due to surface tension. In the experiments the capillary number varies between 0.03 and 0.003. Thus, unless otherwise specified we chose $Ca = 0.01$.
The dimensionless numbers Q_0^* and Q_2^* give the relative magnitude of monopolar and quadrupolar deformations amplitudes to the particle size. The amplitude of the monopolar deformation, Q_0^*, can reach values up to 0.1, see Supplementary Figure 6c. The amplitude of the quadrupolar deformation, Q_2, varies over a smaller range, and its value typically lies between 10 and 30 nm [6, 7]. Hence, unless otherwise specified, we fix $Q_2^* = 0.01$. Likewise, To reproduce typical experimental conditions the surface coverage is considered to be $\Phi = 0.4$, unless otherwise specified.
The dimensionless numbers ΔR^* and R_0^* are geometrical parameters giving the ratio of the bubble to particle sizes and the amplitude of radial deformation relative to the particle size, respectively. These

numbers have been chosen to match typical experimental conditions. We used $R_0^* = 20$, and $\Delta R^* = 1.5$, resulting in small amplitude oscillations of the bubble $\Delta R/R_0 = 0.075$.

Finally, $K_{\rm EV}^*$ and $K_{\rm S}^*$ relate the strength of the excluded volume interactions and of the constraining force, with respect to the viscous force. These two numbers are chosen to be 2000 and 1500 respectively, to prevent overlap between particles and desorption from the interface.

Following those definitions, we can rewrite the kinematic equations in dimensionless form, denoting all the dimensionless quantities with the superscript *:

$$\frac{4\pi}{3}\, St\, \frac{d\mathbf{v}_i^*}{dt^*} = -3\pi\, \mathbf{v}_i^* + \mathbf{F}_{\rm S}^{*^i} + \sum_{j\neq i}\left(\mathbf{F}_{00}^{*^{ij}} + \mathbf{F}_{22}^{*^{ij}} + \mathbf{F}_{02}^{*^{ij}} + \mathbf{F}_{20}^{*^{ij}} + \mathbf{F}_{EV}^{*^{ij}} + \mathbf{F}_{\rm hyd}^{*^{ij}}\right)\,, \tag{34}$$

$$\frac{d\mathbf{r}_i^*}{dt^*} = \mathbf{v}_i^*\,, \tag{35}$$

$$\frac{8\pi}{15} St \frac{d\boldsymbol{\omega}_i^*}{dt^*} = -4\pi\, \boldsymbol{\omega}_i^* + \sum_{j\neq i}\left(\mathbf{T}_{22}^{*^{ij}} + \mathbf{T}_{20}^{*^{ij}}\right)\,, \tag{36}$$

$$\frac{d\mathbf{p}_i}{dt^*} = \boldsymbol{\omega}_i^* \times \mathbf{p}_i\,. \tag{37}$$

With the dimensionless capillary forces and torques now given by:

$$\mathbf{F}_{00}^{*^{ij}} = \frac{Ca^{-1}\, Q_0^{*^2}\, \sin{(t^*)}^2}{2\,\pi\, d^*}\hat{\mathbf{d}}_{ij}\,, \tag{38}$$

$$\mathbf{F}_{22}^{*^{ij}} = \frac{48\,\pi\, Ca^{-1}\, Q_2^{*^2}}{d^{*^5}}\left[\hat{\mathbf{d}}_{ij}\cos{(2\varphi_i + 2\varphi_j)} - \frac{\mathbf{r}_i^*}{|\mathbf{r}_i^*|}\times\hat{\mathbf{d}}_{ij}\sin{(2\varphi_i + 2\varphi_j)}\right]\,, \tag{39}$$

$$\mathbf{F}_{02}^{*^{ij}} = \frac{2\, Ca^{-1}\, Q_0^*\, Q_2^*\sin{(t^*)}}{d^{*^3}}\left[\hat{\mathbf{d}}_{ij}\cos{(2\varphi_j)} - \frac{\mathbf{r}_i^*}{|\mathbf{r}_i^*|}\times\hat{\mathbf{d}}_{ij}\sin{(2\varphi_j)}\right]\,, \tag{40}$$

$$\mathbf{F}_{20}^{*^{ij}} = \frac{2\, Ca^{-1}\, Q_0^*\, Q_2^*\sin{(t^*)}}{d^{*^3}}\left[\hat{\mathbf{d}}_{ij}\cos{(2\varphi_i)} - \frac{\mathbf{r}_i^*}{|\mathbf{r}_i^*|}\times\hat{\mathbf{d}}_{ij}\sin{(2\varphi_i)}\right]\,, \tag{41}$$

$$\mathbf{T}_{22}^{*^{ij}} = -\frac{24\,\pi\, Ca^{-1}\, Q_2^{*^2}}{d^{*^4}}\frac{\mathbf{r}_i^*}{|\mathbf{r}_i^*|}\sin{(2\varphi_i + 2\varphi_j)}\,, \tag{42}$$

$$\mathbf{T}_{20}^{*^{ij}} = -\frac{2\, Ca^{-1}\, Q_0^*\, Q_2^*\sin{(t^*)}}{d^{*^2}}\frac{\mathbf{r}_i^*}{|\mathbf{r}_i^*|}\sin{(2\varphi_i)}\,. \tag{43}$$

The dimensionless hydrodynamic force is given by:

$$\mathbf{F}_{\rm hyd}^{*^{ij}} = -\beta\,\frac{Q_0^{*^2}}{d^{*^2}}\,\hat{\mathbf{d}}_{ij}\,. \tag{44}$$

The dimensionless excluded volume and the surface trapping forces are given by:

$$\mathbf{F}_{\rm EV}^{*^{ij}} = -2K_{\rm EV}^*\,\delta^*\,\hat{\mathbf{d}}_{ij} \qquad \text{if} \quad \delta^* < 0\,, \tag{45}$$

and

$$\mathbf{F}^{*^{ij}}_{\mathrm{EV}} = 0 \qquad \text{if} \quad \delta^* > 0\,, \tag{46}$$

with δ^* now being $\delta^* = d^* - 2$.

$$\mathbf{F}^{*^{i}}_{\mathrm{S}} = -K^*_{\mathrm{S}} \left(|\mathbf{r}^*_i| - R^*_0 - \Delta R^* \sin(t^*)\right) \frac{\mathbf{r}^*_i}{|\mathbf{r}^*_i|}\,. \tag{47}$$

The simulation presented in Figure 2 of the main paper and in Supplementary Movie 5 starts with an equilibration time (only static interactions are allowed) until a steady configuration is reached. After this, all the forces (static and dynamic) are allowed for 1000 cycles of oscillations. We performed numerical simulations varying Q^*_0 and β to find the values giving final states that are comparable to those observed in the experiments. In the next section we present results of additional simulations to illustrate the effect of individual interactions.

3.7 Effect of individual interactions on the microstructure

To highlight the mechanism driving the formation of strings observed in the experiments, we performed numerical simulations enabling only one type of interaction at a time. We simulate the evolution of the particle positions and orientations for 1000 cycles starting from random configurations. The parameters used are those specified in Section 3.6, except for the surface coverage, which is $\Phi = 0.3$.

Monopole-monopole

The result obtain after 1000 cycles of oscillations when allowing monopole-monopole interactions only is presented in Figure 5a of the main paper. The microstructure obtained consists in a large cluster containing all the particles. This is coherent with the experimental observations of the Cheerios effect where heavy particles imparting a monopolar deformation on the interface aggregate [5]. Changing the parameters in this set of simulations affects the dynamics of the system only slightly. Overall, the resulting structure is always the same.

Quadrupole-quadrupole

When we allow in the simulations only quadrupole-quadrupole interactions for 1000 cycles, we observe as a final structure different clusters of particles on the surface of the bubble. In each cluster, the orientation vectors of the particles are parallel as can be seen in Figure 5b of the main paper. This is coherent with what was previously reported in the literature [9].

Monopole-quadrupole

The cross-interaction between a monopolar deformation and a quadrupolar deformation has the directionality required for the formation of strings. Particles interacting through monopole-quadrupole interaction tend to align their orientation vectors with the center-to-center vector, resulting in a dipolar symmetry which allows for the creation of strings. This was confirmed through simulations performed considering only monopole-quadrupole interactions between particles. The shape of the interface corresponding to that configuration is shown in the inset of Supplementary Figure 5c. The induced capillary force is directional and can lead to either attraction or repulsion depending on the orientation of the particle (green arrow) compared to the center-to-center vector (dashed line). The resulting structure (shown in Figure 5c of the main paper) is a network of strings with a striking similarity to the experimental configurations. It can also be pointed out that the particles tend to align there orientation vectors inside a chain.

Hydrodynamic interactions

In the case of particles interacting only through hydrodynamic forces, the simulations predict the formation of an ordered lattice on the surface of the bubble, see Figure 5d of the main paper. This is expected as repulsive interactions are known to promote the formation of crystals on closed surfaces [20, 21, 22]. This structure was never observed in the experiments.

3.8 Relaxation of the microstructure

We performed simulations of the relaxation of the monolayer after the forcing stops. We started a simulation from a random configuration and we let it evolve during the oscillations of the bubble allowing all the interactions. After 1000 cycles, the oscillations are stopped, the monopole amplitude is set to zero and we study the relaxation of the monolayer due to quadrupolar capillary interactions. Brownian forces are neglected because the energy due to quadrupolar capillary interactions is much larger than the thermal energy $Q_2^2\gamma \approx 10^3 k_B T$. The results are presented in Supplementary Figure 9. As can be seen from the snapshots in Supplementary Figure 9a, the relaxation of the structure is observed 30 ms after the forcing stops. A confirmation of the relaxation of the string structure after 30 ms is given by the fact that the peak at $r = 4a$ in the pair-correlation function is no longer present (Supplementary Figure 9b). Furthermore, the dynamics of relaxation is tracked by observing the slow evolution of the mean number of neighbours $\bar{n}$ in Supplementary Figure 9c.

Scaling laws can be obtained for the rotational and translational relaxation times of a single particle by equating the capillary torque and force with the viscous drag. Both the rotational and translational

drag are approximated with half of Stokes' drag for particles at a liquid-gas interface. The torque between two particles at contact due to quadrupolar interactions, T_{22}, balances the rotational drag $\zeta_{\mathrm{r}} \approx 4\pi\eta a^3\dot{\varphi}$, giving a characteristic rotational velocity $\dot{\varphi} \approx 3Q_2^2\gamma/(8\eta a^3)$. The rotational relaxation time can be estimated as the time to rotate of an angle π: $\tau_{\mathrm{r}} \approx 8\pi\eta a^3/(3Q_2^2\gamma)$. Using the typical values from the simulations for viscosity, particles radius, quadrupole amplitude and surface tension, we find a rotational relaxation time $\tau_{\mathrm{r}} \approx 10$ ms. The quadrupole-quardupole interaction force, F_{22}, between two particles at contact balances the translational drag $\zeta_{\mathrm{t}} \approx 3\pi\eta a V$ to give a typical translational velocity $V \approx Q_2^2\gamma/(2\eta a^2)$. The translational relaxation time can be estimated as the time for a particle to move by a distance a: $\tau_{\mathrm{t}} \approx 2\eta a^3/(Q_2^2\gamma)$. Using the typical values for the parameters, we find a translational relaxation time, $\tau_{\mathrm{t}} \approx 5$ ms.

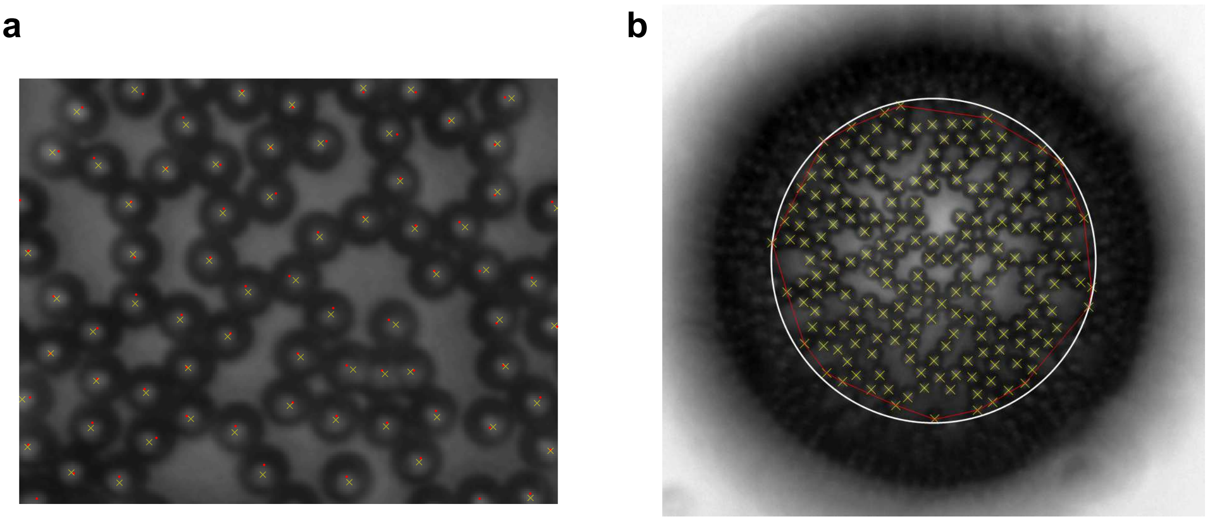

Supplementary Figure 1: **Particle detection and calculation of surface coverage.** **a** After a manual rough tracking (red dots), an optimisation algorithm is ran to find particle centers with sub-pixel accuracy (yellow crosses). **b** The density is computed as the mean density between the minimum bounded circle (white circle) and the convex hull (red polygon).

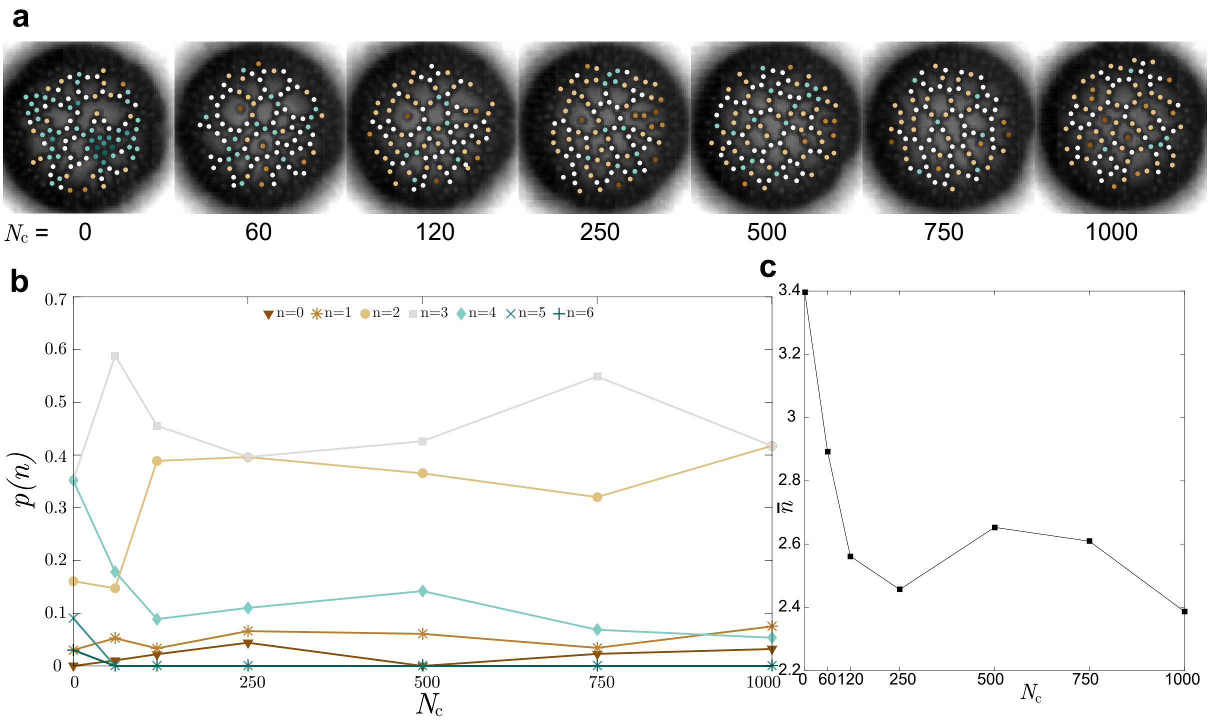

Supplementary Figure 2: **Characterisation of the evolution of the microstructure for the experiment in Figure 2 of the main paper**. **a** Images during 1000 cycles of bubble oscillations, with particles colour-coded according to number of neighbours. **b** Evolution of the probability $p(n)$ of having a number of neighbours equal to n. **c** Evolution of the mean number of neighbours $\bar{n}$ during bubble oscillations.

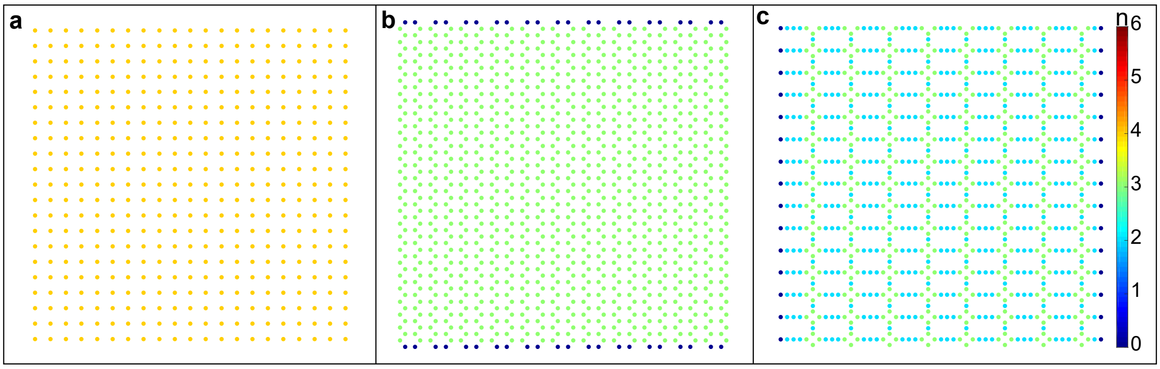

Supplementary Figure 3: **Simulated ideal networks with different bond order parameters.** **a** $|\Psi_2| = 1$, $|\Psi_3| = 0$. **b** $|\Psi_2| = 0$, $|\Psi_3| = 1$. **c** A branched network of particles with $|\Psi_2| = 0.67$ and $|\Psi_3| = 0.40$.

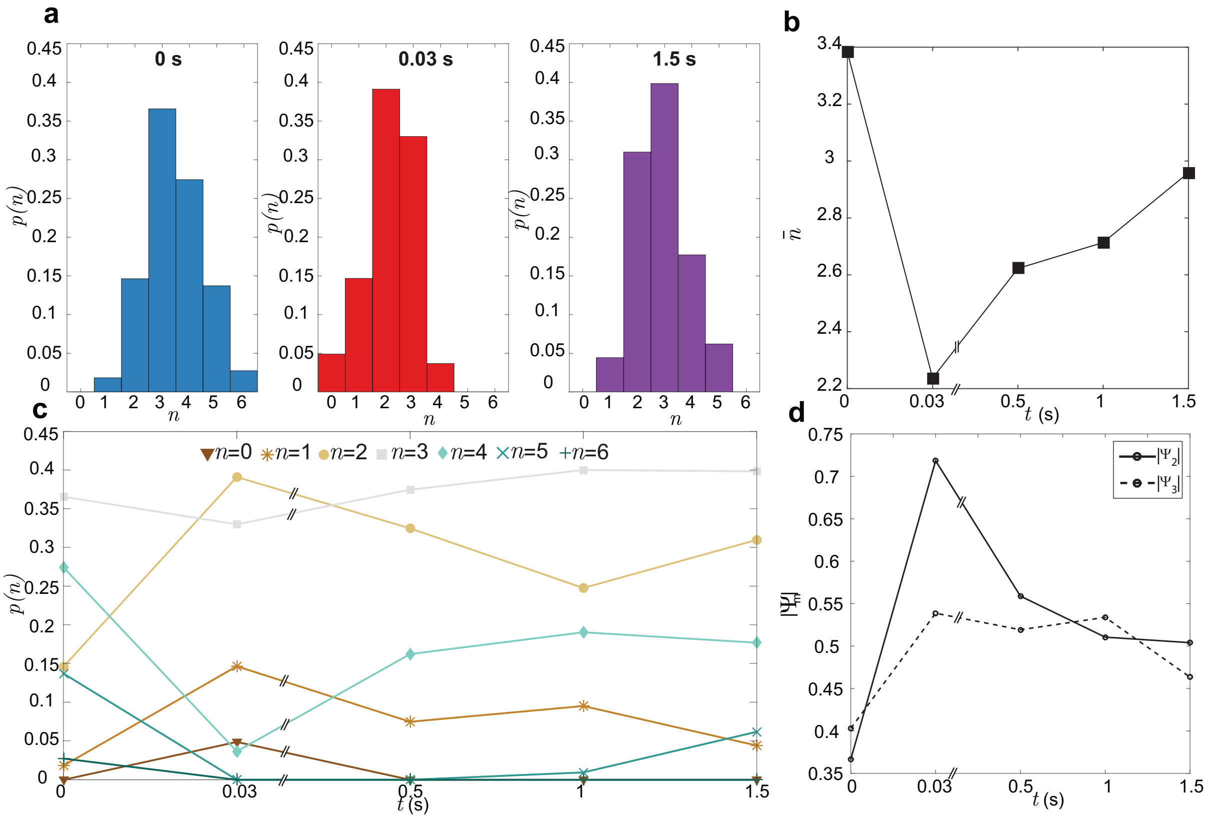

Supplementary Figure 4: **Analysis of relaxation data for the experiment in Figure 1c of the main paper**. The relaxation starts after 1000 cycles ($t > 0.03$ s). **a** Histograms of $p(n)$ at three different times, initial ($t = 0$ s), after the 1000 oscillations ($t = 0.03$ s), during the relaxation process ($t = 1.5$ s). **b** Evolution of the mean number of neighbours during the relaxation process ($t > 0.03$ s). **c** Evolution of $p(n)$ during the relaxation process. **d** Evolution of the bond order parameters $|\Psi_2|$ and $|\Psi_3|$ during relaxation.

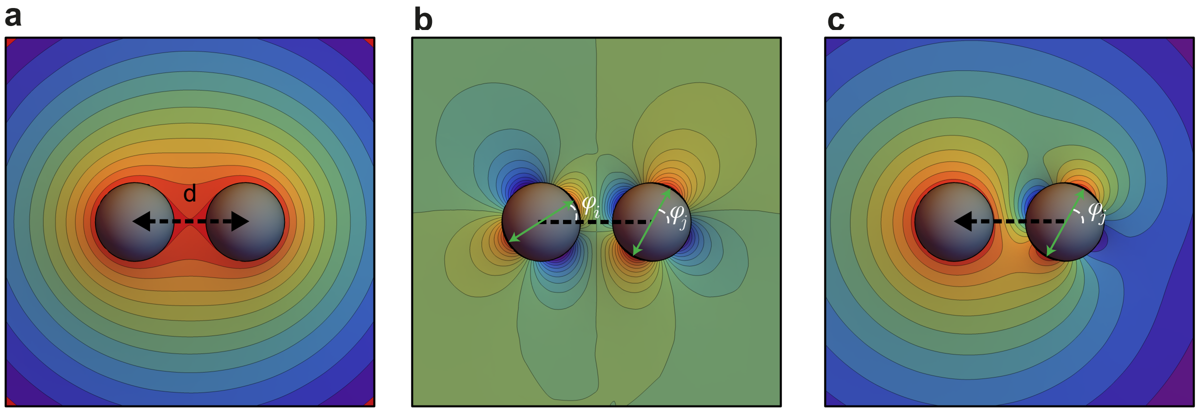

Supplementary Figure 5: **Modes of interface deformation around pairs of particles.** **a** Superposition of two monopolar deformations at a distance d. The interaction force is attractive and directed along the center-to-center vector. **b** Superposition of two quadrupolar deformations. The interaction force depends on the center-to-center distance d and on the particle orientations φ_i and φ_j. **c** Superposition of a monopolar deformation and a quadrupolar deformation. The interaction between the two particles depends on the center-to-center distance d and on the angle φ_j.

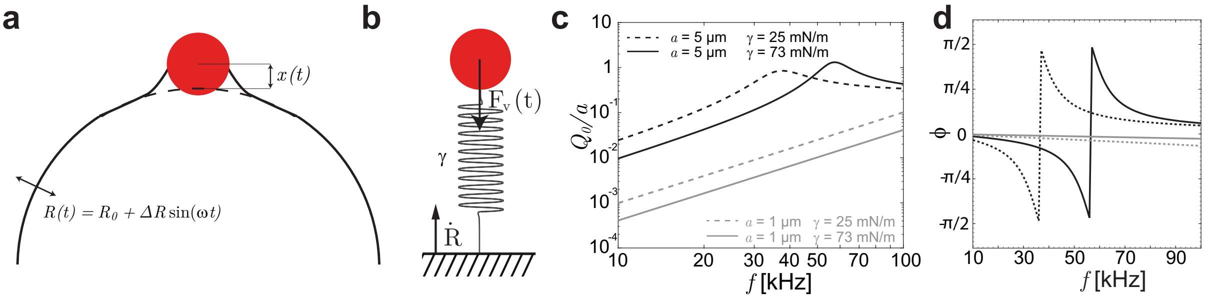

Supplementary Figure 6: **Harmonic oscillator model.** **a**: Schematic of the particle deforming the interface. $x(t)$ is the position of the particle centre with respect to the undeformed interface, which has position $R(t)$. **b**: Schematic of a particle held to interface by a spring of constant γ, generating a restoring force $F_v(t) = -\gamma x(t)$. **c**: Amplitude of the deformation Q_0/a as a function of the frequency $f = \omega/2\pi$. Four extreme scenarios are plotted; $a = 1$ μm (gray) and $a = 5$ μm (black); $\gamma = 73$ mN m^{-1} (solid lines) and $\gamma = 25$ mN m^{-1} (dashed lines). **d**: Phase difference ϕ between the particle and the interface oscillations as a function of frequency, for the four scenarios of part **c**.

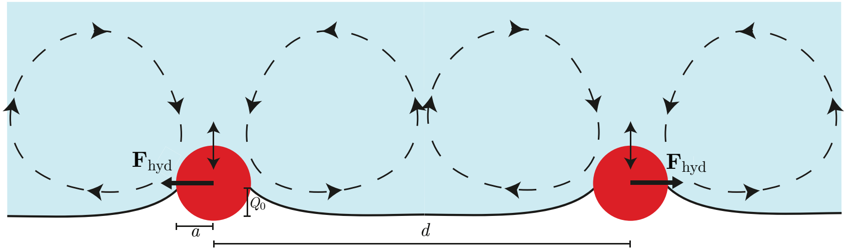

Supplementary Figure 7: **Hydrodynamic interactions between particles oscillating normal to a fluid interface**. The oscillation of amplitude Q_0 of the particles around the interface generates steady streaming flows resulting in a repulsive interaction force $\mathbf{F}_{\rm hyd}$.

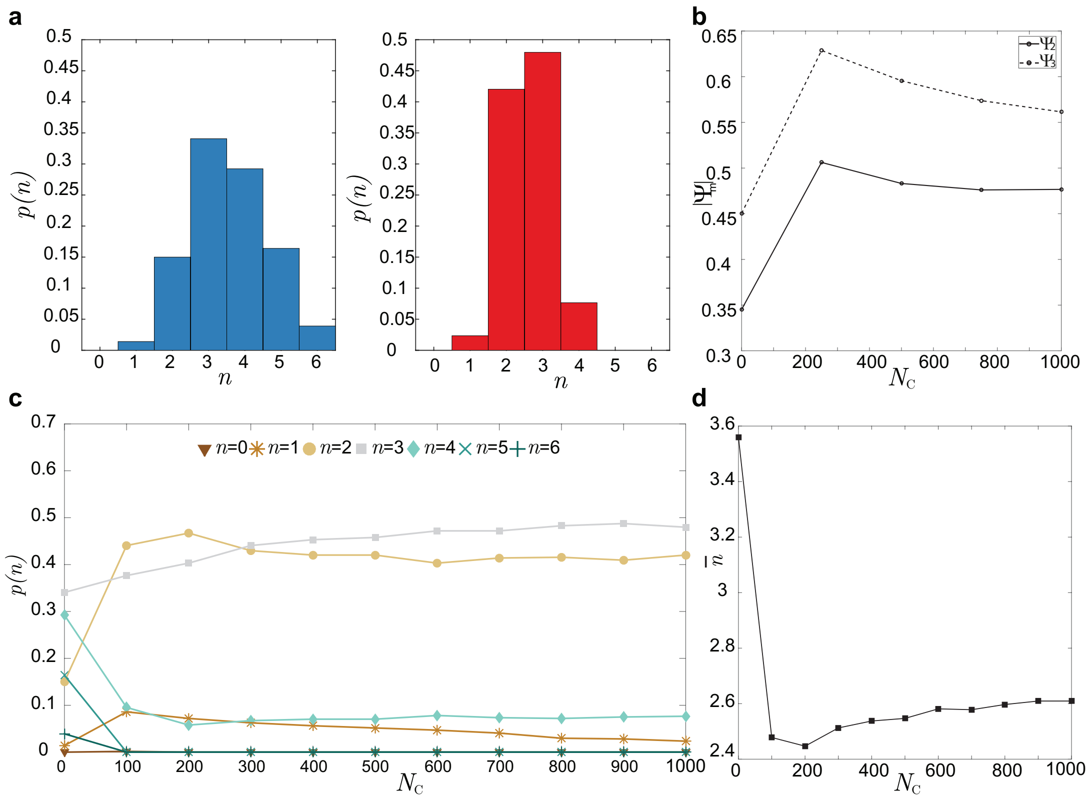

Supplementary Figure 8: **Characterisation of the evolution of the microstructure for the simulations in Figure 2 of the main paper. a** Histograms of the probability of having n neighbours for the initial state (left panel) and the final state (right panel). **b** Evolution of the bond order parameters Ψ_2 (solid line) and Ψ_3 (dashed line). **c** Evolution of the probability $p(n)$ of having n neighbours during the oscillations. **d** Evolution of the mean number of neighbours $\bar{n}$ during the oscillations.

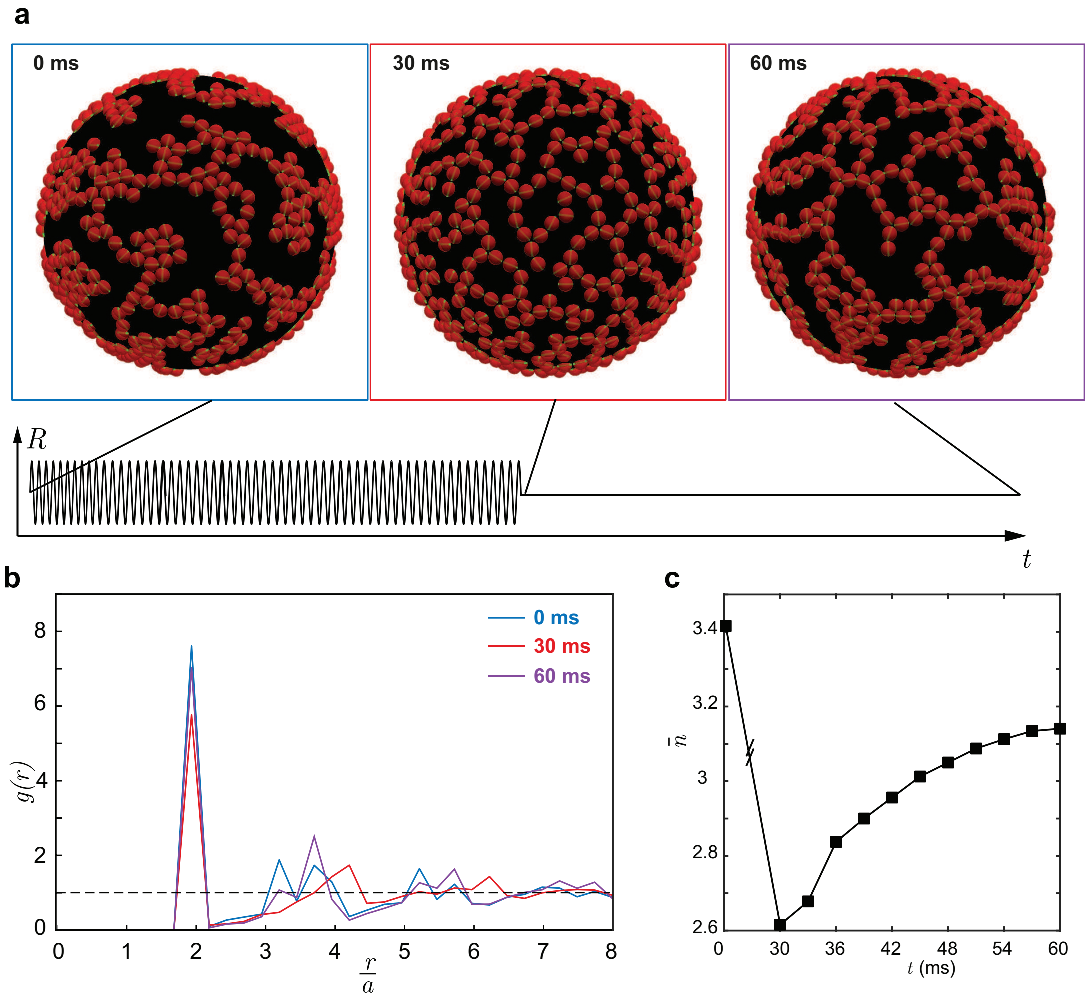

Supplementary Figure 9: **Characterisation of the relaxation of the microstructure for the same parameters as the simulations in Figure 2 of the main paper.** **a** Snapshots of the microstructure before the oscillations (0 s), after them (0 .03 s) and after a 0.03 s of relaxation. **b** Evolution of the pair-correlation function $g(r)$ at time t= 0 s (blue), 0.03 s (red) and 0.06 s (purple) **c** Evolution of the mean number of neighbours $\bar{n}$ during the relaxation period (oscillations stopped at 30 ms).

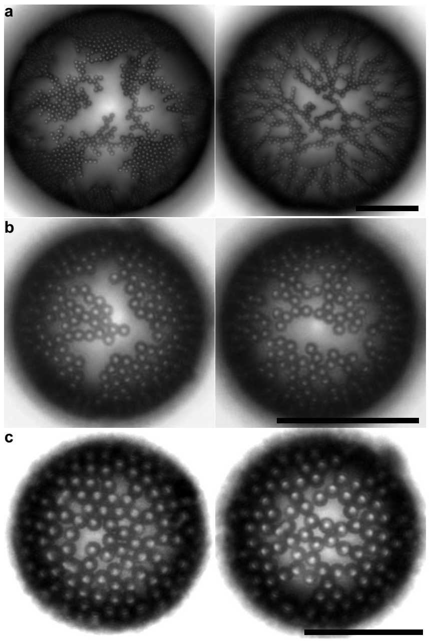

Supplementary Figure 10: **String formation for different surface coverages.** Left frames: initial state. Right frames: final state. **a** $\Phi = 0.38$. **b** $\Phi = 0.49$. **c** $\Phi = 0.38$. All scale-bars: $40\,\mu$m.

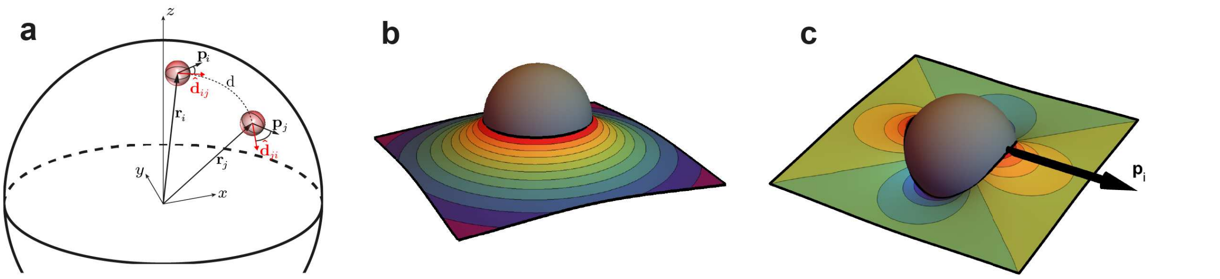

Supplementary Figure 11: **Notations for the interaction model**. **a** Schematic of two particles adsorbed on the bubble. **b** Monopolar deformation of the interface. **c** Quadrupolar deformation. The particle is given an orientation $\mathbf{p_i}$. In both **b** and **c**, the colour scale represents a hight of the interface above the unperturbed position (red) or below (blue). The deformations are exaggerated for clarity.

Supplementary References

[1] Parthasarathy, R. Rapid, accurate particle tracking by calculation of radial symmetry centers. *Nat. Methods* **9**, 724–726 (2012).

[2] Pitois, O. & Chateau, X. Small particle at a fluid interface: Effect of contact angle hysteresis on force and work of detachment. *Langmuir* **18**, 9751–9756 (2002).

[3] Domínguez, A., Oettel, M. & Dietrich, S. Force balance of particles trapped at fluid interfaces. *J. Chem. Phys.* **128**, 114904 (2008).

[4] Danov, K. D., Kralchevsky, P. A., Naydenov, B. N. & Brenn, G. Interactions between particles with an undulated contact line at a fluid interface: Capillary multipoles of arbitrary order. *J. Colloid Interface Sci.* **287**, 121–134 (2005).

[5] Vella, D. & Mahadevan, L. The "Cheerios effect". *Am. J. Phys.* **73**, 817–825 (2005).

[6] Sharifi-Mood, N., Liu, I. B. & Stebe, K. J. Curvature capillary migration of microspheres. *Soft Matter* **11**, 6768–6779 (2015).

[7] Zanini, M. *et al.* Universal emulsion stabilization from the arrested adsorption of rough particles at liquid-liquid interfaces. *Nat. Commun.* **8**, 15701 (2017).

[8] Danov, K. D. & Kralchevsky, P. A. Capillary forces between particles at a liquid interface: General theoretical approach and interactions between capillary multipoles. *Adv. Colloid Interface Sci.* **154**, 91–103 (2010).

[9] van Nierop, E. A., Stijnman, M. A. & Hilgenfeldt, S. Shape-induced capillary interactions of colloidal particles. *EPL (Europhysics Letters)* **72**, 671 (2005).

[10] Park, B. J. & Furst, E. M. Attractive interactions between colloids at the oil–water interface. *Soft Matter* **7**, 7676–7682 (2011).

[11] Riley, N. On a sphere oscillating in a viscous fluid. *Quart. J. Mech. Appl. Math.* **19**, 461–472 (1966).

[12] Riley, N. Steady streaming. *Ann. Rev. Fluid Mech.* **33**, 43–65 (2001).

[13] Otto, F., Riegler, E. K. & Voth, G. A. Measurements of the steady streaming flow around oscillating spheres using three dimensional particle tracking velocimetry. *Phys. Fluids* **20**, 093304 (2008).

[14] Voth, G. A. *et al.* Ordered clusters and dynamical states of particles in a vibrated fluid. *Phys. Rev. Lett.* **88**, 234301 (2002).

[15] Klotsa, D., Swift, M. R., Bowley, R. M. & King, P. J. Interaction of spheres in oscillatory fluid flows. *Phys. Rev. E* **76**, 056314 (2007).

[16] Klotsa, D., Swift, M. R., Bowley, R. M. & King, P. J. Chain formation of spheres in oscillatory fluid flows. *Phys. Rev. E* **79**, 021302 (2009).

[17] Poulichet, V. & Garbin, V. Ultrafast desorption of colloidal particles from fluid interfaces. *Proc. Natl. Acad. Sci. USA* **112**, 5932–5937 (2015).

[18] Singh, P., Joseph, D. D., Gurupatham, S. K., Dalal, B. & Nudurupati, S. Spontaneous dispersion of particles on liquid surfaces. *Proc. Natl. Acad. Sci. USA* **106**, 19761–19764 (2009).

[19] Dörr, A., Hardt, S., Masoud, H. & Stone, H. A. Drag and diffusion coefficients of a spherical particle attached to a fluid–fluid interface. *J. Fluid Mech.* **790**, 607–618 (2016).

[20] Reynaert, S., Moldenaers, P. & Vermant, J. Control over colloidal aggregation in monolayers of latex particles at the oil- water interface. *Langmuir* **22**, 4936–4945 (2006).

[21] Ershov, D., Sprakel, J., Appel, J., Stuart, M. A. C. & van der Gucht, J. Capillarity-induced ordering of spherical colloids on an interface with anisotropic curvature. *Proc. Natl. Acad. Sci. USA* **110**, 9220–9224 (2013).

[22] Gu, C. & Botto, L. Direct calculation of anisotropic surface stresses during deformation of a particle-covered drop. *Soft Matter* **12**, 705–716 (2016).
