# Supplementary material for: Dynamic capillary assembly of colloids at interfaces with 10,000g accelerations
Source: Nat Commun. 2018 Sep 6;9:3620. doi: 10.1038/s41467-018-06049-9 (PMC6127265; doi:10.1038/s41467-018-06049-9)
Supplement: Supplementary file 3 — Description of Additional Supplementary Files [file 41467_2018_6049_MOESM3_ESM.pdf]

<u>Legends for supplementary movies</u>

Supplementary Movie 1

High-speed video of monolayer oscillations at 40 kHz shown for 100 cycles after strings have formed. The bubble is coated with 5-μm particles at a surface coverage Φ = 0.48 and the amplitude of oscillations is small ($\Delta R/R_0$ = 0.047).

Supplementary Movie 2

Stroboscopic video during 1000 cycles of oscillations at 40 kHz, showing the formation of strings after a few tens of cycles. The bubble is coated with 5-μm particles at a surface coverage Φ = 0.48 and the amplitude of oscillations is small ($\Delta R/R_0$ = 0.047).

Supplementary Movie 3

High-speed video of an experiment where no evolution of the microstructure is observed during driving at 40 kHz. The bubble is coated with 5-μm particles at a surface coverage Φ ~ 0.9. The oscillations are small ($\Delta R/R_0$ = 0.045) and the dense network of particles remains unchanged.

Supplementary Movie 4

High-speed video of an experiment where expulsion of particles is observed during driving at 40 kHz. The bubble is coated with 5-μm particles and the amplitude of oscillations is sufficiently large ($\Delta R/R_0$ = 0.1) to drive particle expulsion.

Supplementary Movie 5

Discrete particle simulations using the interaction model including dynamic capillary interactions. The first part of the video shows the initialisation of the monolayer with static interactions. In the second part of the video, dynamic interactions are also included, and the simulation is run for 1000 cycles of oscillations. The video is stroboscopic, so that only the irreversible movements of the particles on the surface are visible, and string formation is immediately apparent. Parameters used in the simulation: Φ=0.4, R_0=20a, $\Delta R/R_0$=0.075, Ca=0.01, Q_0=0.075a, Q_2=0.01a, β = 6π, and St=1.
